# Spatial distribution and determinants of acute respiratory infection among under-five children in Ethiopia: Ethiopian Demographic Health Survey 2016

**DOI:** 10.1371/journal.pone.0215572

**Published:** 2019-04-22

**Authors:** Erkihun Tadesse Amsalu, Temesgen Yihunie Akalu, Kassahun Alemu Gelaye

**Affiliations:** 1 Department of Public health, College of Medicine and Health sciences, Wollo University, Dessie, Ethiopia; 2 Department of Epidemiology and Biostatistics, Institute of Public Health, College of Medicine and Health Sciences, University of Gondar, Gondar, Ethiopia; University of Tennessee Knoxville, UNITED STATES

## Abstract

**Background:**

Childhood acute respiratory infection remains the commonest global cause of morbidity and mortality among under-five children. In Ethiopia, it remains the highest burden of the health care system. The problem varies in space and time, and exploring its spatial distribution has supreme importance for monitoring and designing effective intervention programs.

**Methods:**

A two stage stratified cluster sampling technique was utilized along with the 2016 Ethiopian Demographic and Health Survey (EDHS) data. About 10,006 under-five children were included in this study. Bernoulli model was used to investigate the presence of purely spatial clusters of under-five acute respiratory infection using SaTScan.ArcGIS version 10.1 was used to visualize the distribution of pneumonia cases across the country. Mixed-effect logistic regression model was used to identify the determinants of acute respiratory infection.

**Result:**

In this study, acute respiratory infection among under-five children had spatial variations across the country (Moran’s I: 0.34, p < 0.001). Acute respiratory infection among under-five children in Tigray (p < 0.001) and Oromia (p < 0.001) National Regional States clustered spatially. History of diarrhoea (Adjusted Odds Ratio (AOR) = 4.71, 95% CI: (3.89–5.71))), 45–59 months of age (AOR = 0.63, 95% CI: (0.45–0.89)), working mothers (AOR = 1.27, 95% CI: (1.06–1.52)), mothers’ secondary school education (AOR = 0.65; 95% CI: (0.43–0.99)), and stunting (AOR = 1.24, 95% CI: (1.00–1.54)) were predictors of under-five acute respiratory infection.

**Conclusion and recommendation:**

In Ethiopia, acute respiratory infection had spatial variations across the country. Areas with excess acute respiratory infection need high priority in allocation of resources including: mobilizing resources, skilled human power, and improved access to health facilities.

## Background

Although global child mortality rates have declined consistently over the last decades, preventable infectious diseases continue to require special attention. Infectious diseases are the most significant causes of under-five mortality and accounts for 68% of all deaths among children under the age of five years [1]. According to Global Health Observatory (GHO) 2016 report the leading causes of death among under-five children were preterm birth complications, acute respiratory infections, intrapartum-related complications, congenital anomalies, and diarrhoea [2]. Acute respiratory infection (ARI) is characterized by cough accompanied by short rapid breathing and commonly resulted in death through co-morbidities with other childhood illnesses [3].

Globally, ARI among children remains the second common cause of morbidity and mortality among under-five children. In developing countries including Ethiopia, ARI remains the highest burden [4]. In 2015, it killed around one million children under the age of five years and constitutes 16% of all deaths [5], which is higher than the proportion of deaths from diarrhoea, malaria, and measles combined [6].

About 90% of all ARI among under-five children deaths were reported in the Sub-Saharan Africa and Southeast Asia. Globally, more than 12 million children with severe ARI were admitted to hospitals every year [7]. In Ethiopia, about 3.4 million children suffer from ARI annually and Ethiopia is among the top 15 countries with highest burdens of ARI [8]. It accounts for 18% of all deaths, and kills over 40,000 under-five children every year [9].

The incidence of ARI among under-five children varies among countries at large and regions in particular. These differences can be attributed to child, mother, environment related factors, and co-morbid diseases like: measles, diarrhoea, and malaria [10].

These factors have also favoured the occurrence of ARI cases in clusters or ‘hotspots’ in both high-income [11–13] and low-income countries [14] as well as across geographic settings at both household [14] and regional levels of analyses [15].

Thus, the identification of geographical areas with high burden of disease transmission using geographic information systems (GIS) and spatial statistical analyses has become indispensable to guide targeted interventions. However, past studies in Ethiopia have focused on prevalence and factors associated with the disease [16–18]. The findings of these studies are insufficient and limited to capture geographic patterns of the disease. Therefore, the current study was done to explore the spatial distribution and determinants of ARI among under-five children in Ethiopia using geographic analysis techniques.

## Method and materials

### Study design, setting, and period

**Fig 1:**
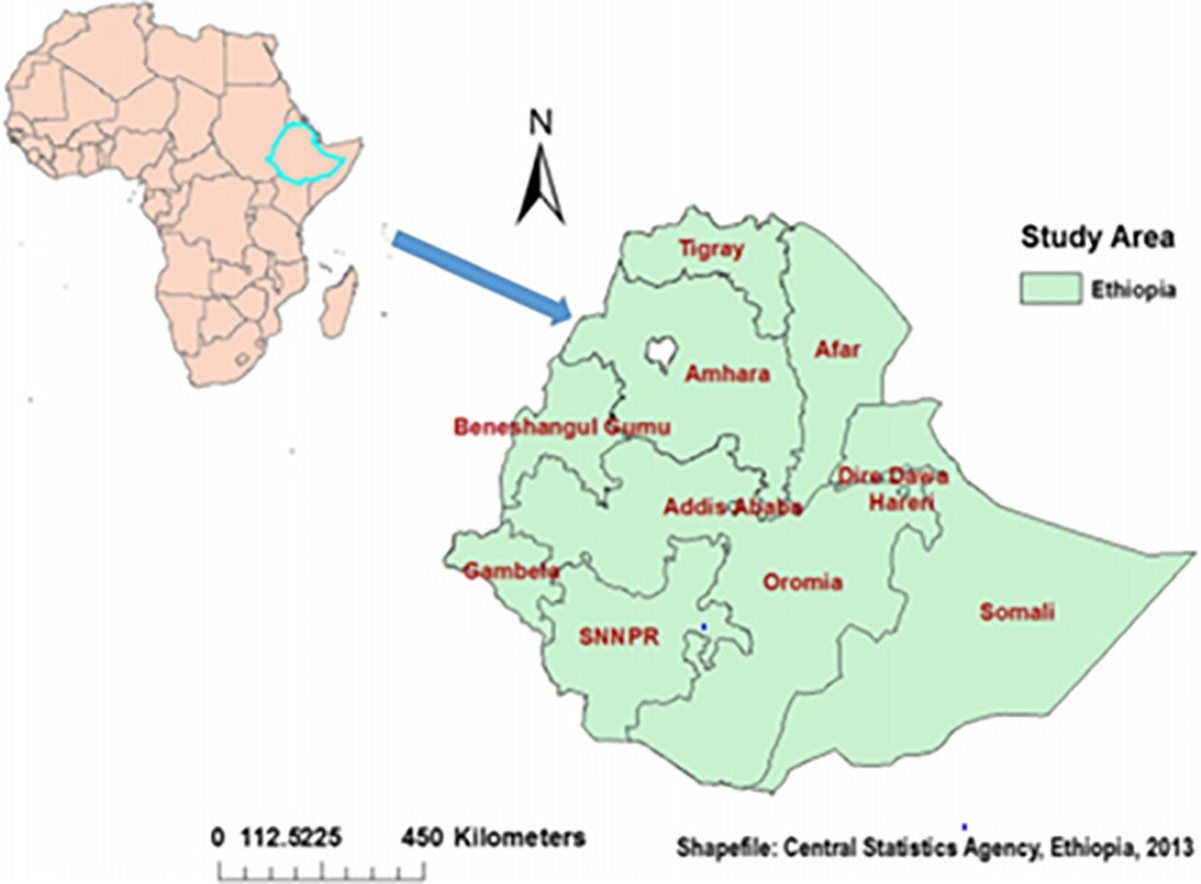
Map of the study area.

A community-based cross-sectional study was conducted from January 18 to June 27, 2016. The study was conducted in Ethiopia (3^o^-14^o^ N and 33^o^ - 48°E), situated in the eastern tip of Africa **(Fig 1)**. The country covers an area of 1.1 million km^2^ (square kilometre) with geographical diversity, ranging from 4,550 meter (m) above sea level down to the Afar depression 110 m below sea level. There are nine regional states and two city administrations sub-divided into 68 zones, 817 districts, and 16,253 kebeles (lowest local administrative units of the country) in the administrative structure of the country [19].

### Population and sample

The source population was all under-five children (10,006) included in the 2016 EDHS.A stratified two-stage cluster sampling procedure was employed where enumeration areas (EAs) were the sampling units for the first stage and households were the second stage. In the 2016 EDHS, a total of 645 EAs (202 urban and 443 rural) were selected with a probability proportional to EAs size (based on the 2007 housing and population census) and independent selection in each sampling stratum. Of these, 18,008 households and 16,583 eligible women were included. The detailed sampling procedure was presented in the full EDHS report [19].

### Data collection procedures and variables

Acute respiratory infection among under-five children two weeks prior to data collection were used as a dependent variable. The independent variables were classified as socio-demographic factors including sex, age of child, residence, maternal education, maternal occupation, number of under-five children, religion, and wealth index; environmental factors were source of water, types of toilet facility, and types of cooking fuel. Clinical factors including vaccination history and drug for intestinal parasites, nutritional factors: breast feeding duration, stunting, wasting, and vitamin A supplement, and co-morbidity conditions including history of diarrhoea, HIV status, anaemia status, and measles.

Acute respiratory infection was defined as children that had history of cough, accompanied by short rapid breathing and/ or difficulty of breathing reported by mothers or caregivers within two weeks preceding the survey [20, 21].Co-morbidity was defined as the presence of one or more additional diseases co-occurring with a primary disease, pneumonia in under- five children [18, 22].

A letter of approval for the use of the data was secured from the Measure DHS and the data set was downloaded from the website www.measuredhs.com(https://dhsprogram.com/data/available-datasets.cfm). We used EDHS 2016 child data set and extracted the outcome and explanatory variables. Location data (latitude and longitude coordinates) was also taken from selected enumeration areas (clusters).

A structured and pre-tested questionnaire was used as a tool for data collection. The 2016 EDHS interviewers used tablet computers to record responses during interviews. The tablets were equipped with Bluetooth technology to enable remote electronic transfer of files (transfer assignment sheets from team supervisors to interviewers and transfer of completed copies from interviewers to supervisors) [19].

### Data management and analysis

Prior to the actual data collection, interviewers were trained and a pre-test was performed. Interviews were performed using local languages [19]. Cross tabulations and summary statistics were used to describe the study population. Descriptive and summary statistics were done using STATA version 14 software. In EDHS data, children within a cluster may be more similar to each other than children in the rest of the country. This violates the assumption of traditional regression model which are the independence of observations and equal variance across clusters. This implies that the need to consider the between-cluster variability using advanced models. Therefore, a multilevel model (both fixed and random effects) was used. As the response variable was dichotomous, logistic regression and Generalized Linear Mixed models (GLMM) were fitted. Model comparison was done based on Akakie Information Criteria (AIC), Bayesian information Criteria (BIC), and intra cluster correlation (ICC) values. The model with the lowest AIC was chosen. Variables with ≤0.2 p-values in the bi-variable analysis were fitted in the multivariable model. Adjusted Odds Ratio (AOR) with a 95% Confidence Interval (CI) and p-value <0.05 in the multivariable model were used to declare significant association with ARI. Goodness of fit was checked using deviance and ICC.

### Spatial autocorrelation analysis

Spatial autocorrelation (Global Moran’s I) statistic measure was used to evaluate whether disease patterns were dispersed, clustered, or randomly distributed in the study area. Moran’s I values close to−1 indicated disease dispersed, whereas I close to +1 indicated disease clustered, and disease distributed randomly if I value was zero. A statistically significant Moran’s I (p < 0.05) led to the rejection of the null hypothesis and indicated the presence of spatial autocorrelation. ArcGIS version 10.1 was used for doing the Moran I analysis.

### Spatial scan statistical analysis

Spatial scan statistical analysis was employed to identify the geographical locations of statistically significant spatial clusters of ARI among under-five children using Kuldorff’sSaTScan version 9.4 software [23]. Spatial scan statistic used a scanning window that moves across the study area. Children with ARI were taken as cases and those without the disease as controls to fit the Bernoulli model. The number of cases in each location had Bernoulli distribution and the model required data with or without the disease. The default maximum spatial cluster size of <50% of the population was used as an upper limit, allowing both small and large clusters to be detected, and ignored clusters that contained more than the maximum limit with circular shape of window. A Likelihood ratio test statistic was used to determine whether the number of observed ARI cases within the potential cluster were significantly higher than the expected or not. Primary and secondary clusters were identified using p-values and likelihood ratio tests on the basis of the 999 Monte Carlo replications [24].

### Ethical issues

Ethical clearance was obtained from the University of Gondar Ethical Review Board (IRB). Permission was obtained from Measure DHS International Program which authorized the datasets. All data used in this study were publicly available; and aggregated secondary data with no any personal identity.

## Results

### Socio-demographic characteristics of respondents

A total of 10,641 under-five children were included in the 2016 EDHS survey. Of these, 10,006 of the children were included the analysis. Of 10,006 children, 5,107(51.04%) were males and 8,099 (80.94%), rural dwellers. Regarding maternal educational status, 6,387(63.83%) had no formal schooling. The mean age of the children was 2.4 years or 28.88 months (S.D± 17.45 months). In terms of wealth index, 3,706 (37.04%) of the participants were from the poorest families. About 2,096 (20.95%) of the children were in the age range of 45–59 months **(Table 1)**.

**Table 1:** Socio-demographic characteristics of respondents in Ethiopia from January 18 to June 27, 2016(N = 10,006).

Variable	Frequency(N)	Percent (%)
**Residence** Urban Rural	1,907 8,099	19.06 80.94
**Age of child**		
< 6 months 6–11 months 12–23 months 24–35 months 36–47 months 48–59 months	1,109 1,016 1,929 1,926 1,930 2,096	11.08 10.15 19.28 19.25 19.29 20.95
**Sex of child**		
Male Female	5,107 4,899	51.04 48.96
**Mother Educational level**		
No education Primary Secondary Higher	6,387 2,538 697 384	63.83 25.36 6.97 3.84
**Wealth Index**		
Poorest Poorer Middle Richer Richest	3,706 1,670 1,386 1,227 2,017	37.04 16.69 13.85 12.26 20.16
**Mother Occupation**		
Not working Working	5,920 4,086	59.16 40.84
**Religion**		
Orthodox Protestant Muslim Other	2,944 1,761 5,068 233	29.42 17.60 50.65 2.33
**Number of living children**		
1–3 4–6 above 6	5,045 3,549 1,412	50.42 35.47 14.11

### Environmental characteristics of respondents

In this study, 5,935 (59.3%) of the households obtained water from improved sources. The majority, 8,156 (81.5%) used unimproved toilet facilities. Most of them, 8,312 (83.1%) used wood for cooking **(Table 2).**

**Table 2:** Environmental characteristics of the respondents in Ethiopia from January 18 to June 27, 2016 (N = 10,006).

Variable	Frequency(N)	Percent (%)
**Type of cooking fuel**		
Electricity	518	5.18
Charcoal	896	8.95
Wood	8,312	83.07
Others*	280	2.80
Child lives with respondent	9,748	97.42
Elsewhere	258	2.58
**Source of drinking water**		
Improved	5,935	59.31
not improved	3,943	39.41
other**	128	1.28
**Type of toilet facility**		
Improved	1,686	16.85
not improved	8,156	81.51
Other***	164	

Other religion: catholic and traditional

*Other Fuel: Kerosene, straw/shrubs/grass, agricultural crop

**Other water source: not a de jure resident, lake

***Other toilet facility: not a de jure resident, no facility/bush/field

Improved water source: (piped into dwelling, piped to yard/plot, public tap/standpipe, tube well or borehole, protected well, protected spring, bottled water); Improved toilet facility: (flush to piped sewer system, flush to septic tank, flush to pit latrine, ventilated improved pit latrine, pit latrine with slab, composting toilet.

### Nutritional and co-morbid characteristics of respondents

Of the children, 8,916 (89.1%) had no history of diarrhoea. About 7, 517 (75.1%) were not vaccinated against any type of vaccine preventable diseases and 9,622 (96.2%) were never breastfed. Regarding nutritional status, 7,876 (78.7%), were wasted and 6,009 (60.1%) were stunted. The majority, 8,830 (88.3%) of children received no drugs for intestinal parasites in the last 6 months, and 5,626 (56.2%) did not get vitamin A in the same period **(Table 3)**.

**Table 3:** Nutritional and co-morbid characteristics of ARI among under-five children in Ethiopia from January 18 to June 27, 2016 (N = 10,006).

Variable	Frequency (N)	Percent (%)
**Duration of breastfeeding**		
ever breastfed	9,622	96.16
never breastfed	384	3.84
**Currently breastfeeding**		
Yes	6,578	65.74
No	3,428	34.26
**Vitamin A supplement**		
Yes	4,380	43.77
No	5,626	56.23
**Stunting**		
normal	6,009	60.05
moderate	1,614	16.13
severe	2,383	23.82
**Wasting**		
normal	7,876	78.71
moderate	828	8.28
severe	1,302	13.01
**Had diarrhoea**		
Yes	1,090	10.89
No	8,916	89.11
**Drug for intestinal parasites**		
Yes	1,176	11.75
No	8,830	88.25

**Fig 2:**
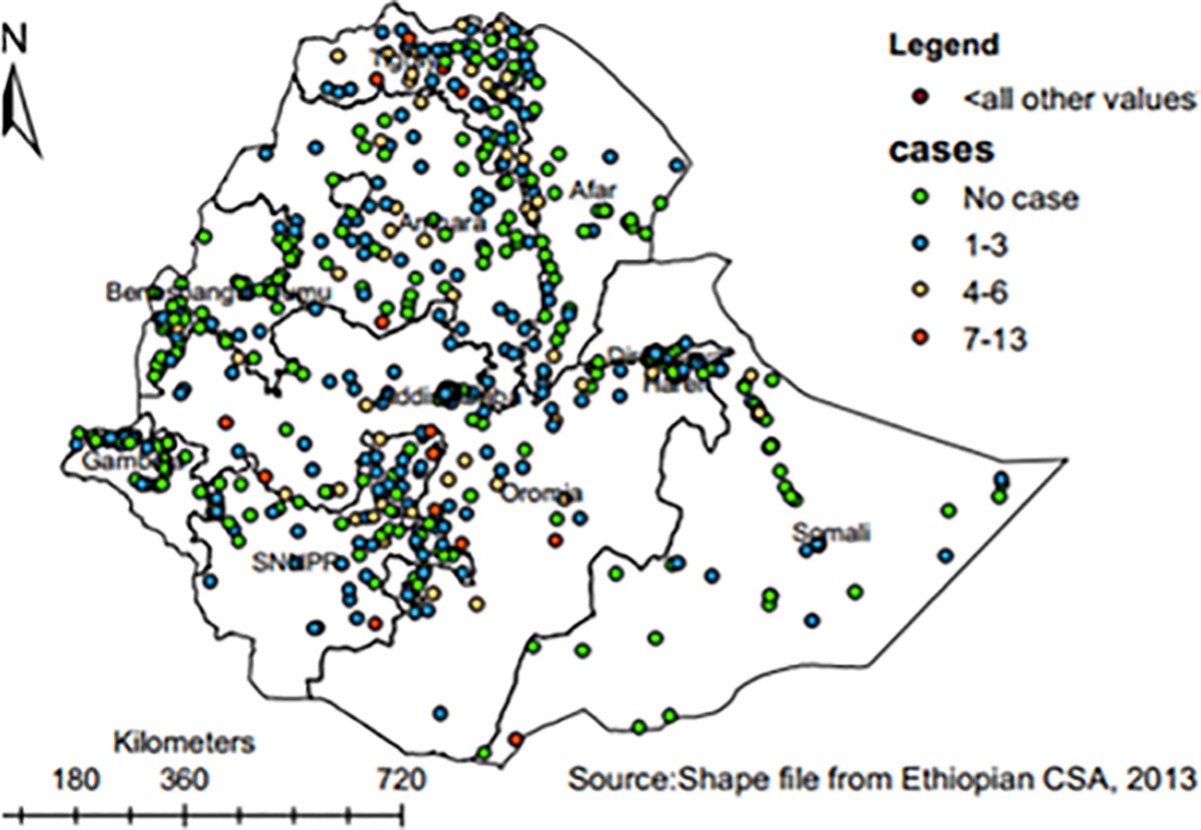
Spatial distribution of ARI across regions in Ethiopia, 2016.

The overall prevalence of ARI among under-five children in Ethiopia was 7.9% (95%CI: 7.4%, 8.5%). Distribution of ARI among under-five children was highest in Tigray(14.92%) and lowest in Benishangul-Gumuz (2.21%) National Regional States.Tigray, Oromia, Amhara, and SNNP National Regional States demonstrated regional variations in the prevalence of under-five ARI **(Fig 2)**.

### Spatial distribution of under-five pneumonia

This study revealed that the spatial distribution of ARI among under-five children was non-random with Global Moran’s I 0.34 (p<0.001). The clustered patterns (on right sides) showed that high rates of ARI occurred across the study area. A z-score of 6.3 indicated that there was less than 1% likelihood for this clustered pattern to be the result of a random chance. The bright red and blue colours at the end tails indicated an increased significance level.

Each spot (point data) on the map represents one census enumeration area which encompasses a number of ARI cases. More cases in the diagram depict ARI hotspot areas. The red colour indicates areas with high rates of ARI cases, whereas the green one indicates low rates.

### Spatial scan statistical analysis

A total of 58 significant clusters were identified at which 22 were most likely (primary) and 36 secondary clusters. The primary clusters were located in Tigray National Regional State (central part). The primary clusters werecentred at 13.769067N, 38.215600E with 85.3 km radius, a relative risk (RR) of 2.95 and Log-Likelihood ratio (LLR) of 38.5, at p<0.001. It showed that children within this area had 3.0 times higher risk for ARI than children outside the area **(Table 4)**.

**Table 4:** Significant spatial clusters of ARI among under-five children in Ethiopia, 2016.

Clusters	Enumeration areas(clusters) detected	Coordinates/radius	Population	Cases	RR	LLR	P- value
1	40, 188, 551, 181, 98, 584, 425, 255, 156, 583, 597, 636, 258, 528, 400, 590, 81, 80, 579, 78, 268, 575	13.769067N,38.215600E)/85.29 km	390	86	2.95	38.53	<0.001
2	555, 558,17,586	8.130989N, 35.637972E)/ 60.43 km	95	28	4.39	22.27	<0.001
3	223, 271, 272, 297, 577, 359, 537, 331, 174, 41, 14, 633, 502, 204, 142, 360, 420, 388, 373, 262, 609, 54, 565, 126, 139, 578, 162, 522, 227, 347, 217, 113	(7.775278N,37.939392E)/95.73km)	589	89	2.17	21.29	<0.001

The two secondary clusters’ were typically located in the border of Oromia National Regional State. The first secondary clusters’ was centred at 8.130989 N, 35.637972 E with 60.4 km radius, RR) of4.39and LLR of 22.3, at p < 0.001. It showed that children within the area had 4times higher risk of ARI than children outside the area. The second secondary clusters’ was centred at 7.775278 N, 37.939392 E within 95.7 km radius, and RR of 2.17 and LLR of21.29, at p < 0.001. It showed that children within the area had 2 times higher risk of ARI than children outside the area.

**Fig 3:**
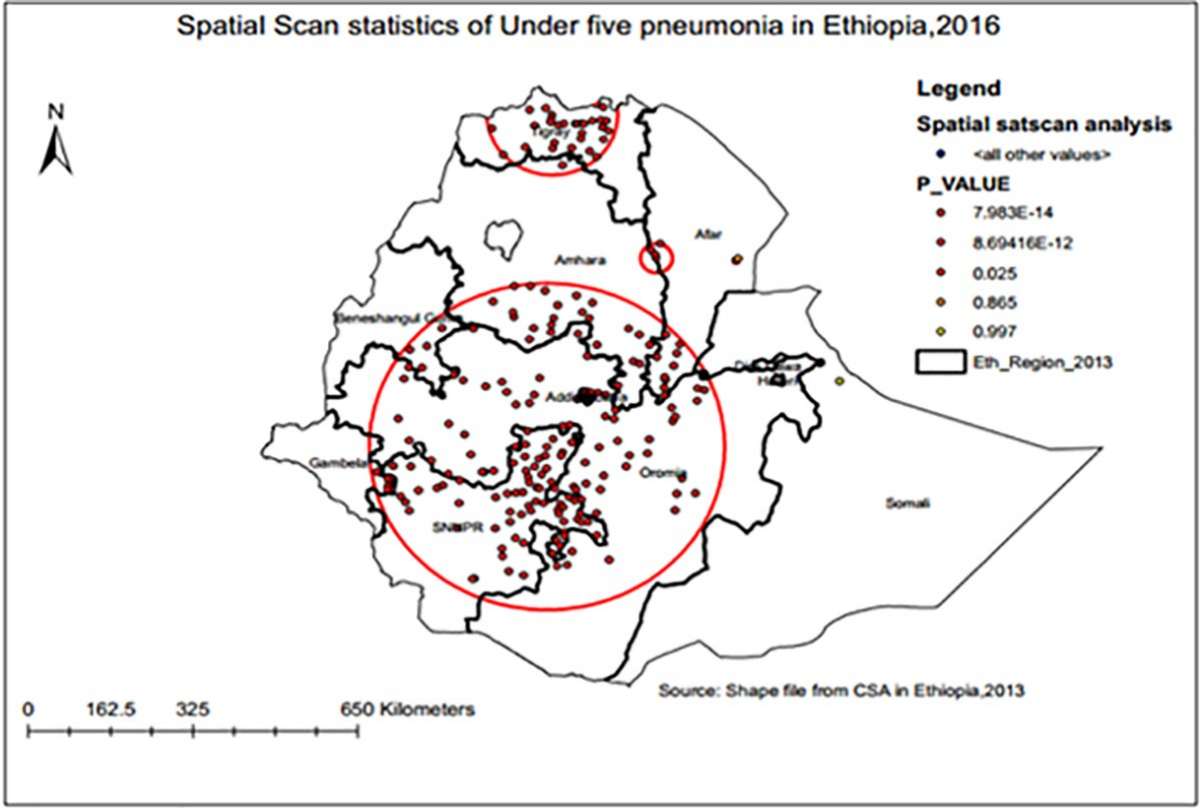
Most likely (primary) and secondary clusters of ARI among under-five children across regions in Ethiopia, 2016.

The bright red colours (rings) indicate that the most statistically significant spatial windows contain primary clusters of ARI. There was a higher risk of ARI within the cluster than outside the cluster **(Fig 3)**.

### Determinants of under-five ARI

#### Model comparison

AIC and BIC were checked, and the mixed effect model was chosen because of the smallest value of AIC **(Table 5)**. Furthermore, the ICC value was 0.2 which informed us to choose GLMM over the basic model.

**Table 5:** Model comparison between logistic regression and mixed effect Logistic regression.

Proposed models	AIC value	BIC value
Logistic regression Mixed effect logistic regression	5178.605 4970.02	5438.199 5258.465

In the mixed effect logistic regression model, number of under-five children in the house, age of child, stunting status, wasting status, residence, maternal educational level, history of diarrhoea in the last two weeks, family wealth index, breastfeeding status, duration of breast feeding, types of fuel for cooking, types of toilet facility, water source for drinking, maternal occupation, history of vaccination, child caretaker, drugs for intestinal parasites in the last six months, and vitamin A supplement in the same period were significant in the bi-variable analysis at p-value <0.05.

However, in the multivariable mixed effect logistic regression analysis, age of child, history of diarrhoea, stunting, maternal education and occupation were significantly associated predictors of ARI among under-five children.

The odds of developing ARI were nearly five times (AOR = 4.7, 95% CI: 3.9, 5.7) higher in children who had diarrhoea compared with their counterparts.

The odds of developing ARI among children age between 48–59 months were decreased by 37% (AOR = 0.63, 95% CI: 0.45, 0. 89) compared with children age less than 6 months.The odds of developing ARI were 24% (AOR = 1.24, 95% CI: 1.01, 1.54) more in moderately stunted children compared with normal ones.

The odds of developing ARI among mothers who completed secondary school were decreased by 35% (AOR = 0.65; 95% CI: 0.43, 0.99) compared with children whose mothers’ had no formal education.The odds of ARI among children who had working mothers were 27% (AOR = 1.27, 95% CI: 1.06, 1.52) higher compared with children whose mothers were not working at the moment **(Table 6)**.

**Table 6:** Bi-variable and Multivariable mixed effect logistic regression analysis of determinants with ARI among under-five children in Ethiopia from January 18 to June 27, 2016 (n = 10,006).

Variable	ARI No Yes		COR (95%CI)	AOR (95%CI)
**Residence**				
Urban Rural	1,799 7,411	108 688	1 1.51 (1.12,2.04)	1 1.28 (0.81,2.04)
**Maternal education**				
No education Primary Secondary Higher	5,872 2,312 661 365	515 226 36 19	1 1.06(0.88,1.27) 0.68(0.47,1.01) 0.66(0.39)	1 0.95(0.77,1.17) 0.65(0.43,0.99) 0.71(0.38,1.24)
**Currently breastfeeding**				
No Yes	3,190 6,020	238 558	1 1.21 (1.02,1.43)	1 0.80 (0.63,1.03)
**Age of child**				
<6 months 6–11 months 12–23 months 24–35 months 36–47 months 48–59 months	1,024 902 1,727 1,774 1,791 1,992	85 114 202 152 139 104	1 1.57 (1.15, 2.16) 1.48 (1.11, 1.96) 1.07 (0.79, 1.44) 0.96 (0.72,1.29) 0.63 (0.46,0.86)	1 1.13(0.82,1.58) 1.01(0.74,1.37) 0.80(0.57,1.12) 0.90(0.65,1.25) 0.63(0.45,0.89)
**Fuel type**				
Electricity Charcoal Wood Other	492 837 7,638 243	26 59 674 37	1 1.40(0.83,2.38) 1.71(1.07,2.72) 2.42(1.33,4.43)	1 1.21(0.69,2.12) 1.19(0.68,2.11) 1.47(0.66,3.27)
**Toilet facility**				
Improved Not improved Other	1,581 7,488 141	105 668 23	1 1.13(0.87,1.46) 1.93(1.12,3.33)	1 0.88(0.65,1.19) 2.23(0.81,6.16)
**Mothers occupation**				
Not working Working	5,506 3,704	414 382	1 1.28(1.09,1.52)	1 1.27(1.07,1.52)
**Wasting status**				
Normal Moderate Severe	7,227 747 1,236	649 81 66	1 1.29(0.99,1.68) 0.68(0.51,0.89)	1 1.21(0.92,1.61) 0.97(0.68,1.38)
**Stunting**				
Normal Moderate Severe	5,514 1,447 2,249	495 167 134	1 1.28(1.05,1.56) 0.71(0.57,0.87)	1 1.24(1.01,1.54) 0.80(0.62,1.05)
**Ever had vaccination**				
No Yes	6,976 2,234	541 255	1 1.49(1.25,1.76)	1 1.20(0.97,1.49)
**Child lives with whom**				
With parents Lives elsewhere	8,960 250	788 8	1 0.39(0.18,0.80)	1 0.64(0.29,1.44)
**Duration of breastfeeding**				
Ever breastfed Never breastfed	8,846 364	776 20	0.60(0.37,0.70) 1	0.75(0.45,1.25) 1

ARI: Acute Respiratory Infection, COR: Crude odds Ratio, AOR: Adjusted odds Ratio

## Discussion

In this study, the distribution of ARI among under-five children varied in the country. The Global Moran’s I value 0.3 (p<0.001) indicated that there was a significant clustering of under-five ARI in the study area.

The Spatial scan statistical analysis identified 58 clusters and allof which were statistically significance. The high risk regions for ARI were the Northern and Central parts of the country mainly Tigray and Oromia. These areas were highland setting implying a high burden of ARI among under-five children. In cold environmental conditions, children are indoors for longer periods and in close contact with a number of bacteria, fungi, or viruses. Flu, viruses, and bacteria are prone to be stable in the air when there is a drop in temperature and the particles remain in the air in respiratory droplets. Bacteria present on our skin are usually harmless; however, during the cold and flu season, immunity decreases weakening the system and even causing damage to the airways creating opportunities for bacteria to cause infections, mainly ARI in the lungs. Another possible reason for this could be a huge number of children were under one year of age, and the majority of the household in these regions used charcoal as a source of fuel [25].

Under-five children who had past history of diarrhoea were more affected by ARI in this study. This finding was supported by a case control study done in Oromia zone, northeast Ethiopia and Zimbabwe where children who had history of diarrhoea were 3 times more likely to develop ARI than their counterparts [16, 26]. Similar studies were done in southwest Ethiopia [27] and Ghana [28]. A Cross sectional survey in Bangladesh [29] also reported that children who had history of diarrhoea were at increased risk for ARI. The possible explanation could be that children who have a concomitant illness like diarrhoea may have a lowered immunity, making them more susceptible to a disease like ARI.

This study showed that working mothers were positively associated with ARI among under- five children. The finding is in line with EDHS 2011 [30], in Pakistan [31], where the risk of developing ARI is significantly higher in children whose mothers were working than children whose mothers were not working. The possible justification could be related to childcare in which mothers play a critical role in childhood ARI[32].The other explanation could be that working mothers have been exposed to certain chemicals, pollutants, or toxic fumes in the working environment, thereby transmitting the infection to their children may be increased. When mothers are at work, they don’t have enough time for breastfeeding their children; thus children become vulnerable to ARI.

Child age was also significantly associated with ARI. The result is consistent with studies conducted in urban areas of Oromia region, Ethiopia [16], where children in the age range of 2–11 months had higher chance of having ARI compared to older age ones. Similarly, a study from Wondo-Genet district, southern Ethiopia [18] younger children (2–12 months) were more likely to develop ARI compared to older age. Similarly, according to the 2013 Lancet report, there was a high occurrence of ARI in children younger than 2 years of age, and a case control study done in Pakistan showed that pneumonia commonly occurred to younger children [31, 33]. Other studies conducted in Indian and the south-eastern regions of Brazil also indicated that children under the age of one year were more likely to be hospitalized for ARI[34, 35]. The possible reason could be that these children build their immune systems over time to fight off infectious agents including ARI.

Stunting was significantly associated with the presence of ARI among under-five children. This result was consistent with those of studies done in South Gondar zone, Ethiopia [17], Bangladesh [36] and Nepal [37]. The possible reason could be that stunting shows a long term malnutrition, which weakens the child’s immunity and makes them vulnerable to ARI. Secondary to Thymo-lymphocyte depletion, malnourished children have defective cell mediated immunity which, leading to infections and sepsis. In addition to this malnutrition also resulted in occurrence of an abnormal immunoglobulin and impairments of key enzymes involved in bactericidal action of leukocytes, which prone to ARI.

In this study, maternal education was found to be significantly associated with ARI. This finding is consistent with those previous studies done in Ethiopia by using EDHS 2011 [30], Bangladesh [36], in developing countries [38], and in Southeast Asia regions [32]. Knowledge that mothers acquire from formal education could help them to adopt essential nutrition and hygienic behaviours that prevent ARI. Another possible reason might be that uneducated mothers compared to educated ones have better health-seeking behaviour which could help them to preventARI.

However, studies done in Este, northwest Ethiopia [17], Wondo-Genet district, southern Ethiopia[18], Debre-Berhan district, northeast Ethiopia (35), Bangladesh [36], and urban eastern Indonesia [39]showed that mothers’ education did not show a significant association. This discrepancy might be due to differences in sample sizes (larger sample size was used in our study).

One of the strengths of this study was its representativeness at national and regional levels, so it can be generalized to all under-five children in Ethiopia. Moreover, the use GIS and Sat Scan statistical tests helped to detect similar and statistically significant high-risk clusters/hotspots of ARI. However, the study didn’t show exact case locations because location data values were shifted 1–2 km for urban and 5km for rural areas for data confidentiality reasons and didn’t take seasonal variations into account of ARI among under-five children. Furthermore, the DHS surveys did not base themselves on clinically confirmed data; rather they relied on mother or caregivers’ reports and might underestimate or overestimate the finding.

The findings of this study have valuable policy implications for health program design and interventions. The ARI hotspot areas can be easily identified to make local interventions. It may also be important to prevent and control ARI outbreaks. In general, these findings are of supreme importance for the Ministry of Health, health Bureaus, and partners to develop intervention programs against under-five ARI.

## Conclusion

In Ethiopia, ARI had spatial variations across the country. Statistically significant high hotspots of ARI among under-five children were found in the northern and central parts of Ethiopia, whereas low hotspots of ARI among under-five children were detected in the western and eastern parts of the country. Stunting, diarrhoea, and maternal working status were predictors that increased the odds of ARI among under-five children. Age of child (45–59 months) and secondary school education of mothers were protective to ARI among under-five children.

## Abbreviations

AIC: Akakie Information Criteria
ARI: Acute Respiratory Tract Infection
AOR: Adjusted Odds Ratio
BIC: Bayesian information criteria
COR: Crude odds ratio
CSA: Central Statics Agency
EAs: Enumeration Area
EDHS: Ethiopian Demographic and Health Survey
GIS: Geographic Information System
GLMM: Generalized Linear Mixed Model
ICC: Intra cluster correlation
SNNP: South Nation Nationality and People
WHO: World Health Organization

## References

[1] Black RE, Cousens S, Johnson HL, Lawn JE, Rudan I, Bassani DG, et al Global, regional, and national causes of child mortality in 2008: a systematic analysis. The lancet. 2010;375(9730):1969–87. 10.1016/S0140-6736(10)60549-1 20466419

[2] http://www.who.int/gho/child_health/mortality/causes/en/.

[3] Johnson W, Abdulkarim A. Childhood pneumonia in developing countries. African Journal of Respiratory Medicine. 2013;8.

[4] UNICEF W. Pneumonia and diarrhoea: Tackling the deadliest diseases for the world’s poorest children. New York: UNICEF 2012:2–8. 10.1016/S0140-6736(12)60907-6 22682449

[5] Organization WH. World health statistics 2015: World Health Organization; 2015.

[6] report Stc. Pneumonia, world's deadliest disease, kills two children every minute. 2017.

[7] UNICF. Pneumonia and diarrhea. New York 2012.

[8] UNICEF. Country estimates of child mortality, causes of under-five deaths and coverage indicators in committing to child survival. 2014.

[9] Prüss‐Ustün A, Bartram J, Clasen T, Colford JM, Cumming O, Curtis V, et al Burden of disease from inadequate water, sanitation and hygiene in low‐and middle‐income settings: a retrospective analysis of data from 145 countries. Tropical Medicine & International Health. 2014;19(8):894–905. 24779548 10.1111/tmi.12329 PMC4255749

[10] Dadi A, Kebede Y, Mengesha Z. Determinants of Pneumonia in Children Aged Two Months to Five Years in Urban Areas of Oromia Zone, Amhara Region, Ethiopia2014. 1–10 p.

[11] Crighton E, Elliott S, Moineddin R, Kanaroglou P, Upshur R. An exploratory spatial analysis of pneumonia and influenza hospitalizations in Ontario by age and gender. Epidemiology & Infection. 2007;135(2):253–61. 16824252 10.1017/S095026880600690X PMC2870578

[12] Crighton EJ, Elliott SJ, Kanaroglou P, Moineddin R, Upshur RE. Spatio-temporal analysis of pneumonia and influenza hospitalizations in Ontario, Canada. Geospatial health. 2008;2(2):191–202. 10.4081/gh.2008.243 18686268

[13] Dias J, Correia A, Queirós L. Community-acquired pneumonia and influenza hospitalisations in northern Portugal, 2000–2005. Euro surveillance: bulletin Europeen sur les maladies transmissibles = European communicable disease bulletin. 2007;12(7):E13–4. 10.2807/esm.12.07.00726-en 17991407

[14] Thörn LK, Minamisava R, Nouer SS, Ribeiro LH, Andrade AL. Pneumonia and poverty: a prospective population-based study among children in Brazil. BMC infectious diseases. 2011;11(1):180. 21696610 10.1186/1471-2334-11-180 PMC3141414

[15] Omer S, Sutanto A, Sarwo H, Linehan M, Djelantik I, Mercer D, et al Climatic, temporal, and geographic characteristics of respiratory syncytial virus disease in a tropical island population. Epidemiology & Infection. 2008;136(10):1319–27. 18177515 10.1017/S0950268807000015 PMC2870725

[16] Dadi AF, Kebede Y, Birhanu Z. Determinants of pneumonia in children aged two months to five years in urban areas of Oromia Zone, Amhara Region, Ethiopia. Open Access Library Journal. 2014;1(08):1.

[17] Fekadu GA, Terefe MW, Alemie GA. Prevalence of pneumonia among under-five children in Este Town and the surrounding rural Kebeles, Northwest Ethiopia: A community based cross sectional study. Science Journal of Public Health. 2014;2(3):150–5.

[18] Abuka T. Prevalence of pneumonia and factors associated among children 2–59 months old in Wondo Genet district, Sidama zone, SNNPR, Ethiopia. Current Pediatric Research. 2017.

[19] International CSAEaI. Ethiopia Demographic and Health Survey 2016. Addis Ababa, Ethiopia, and Calverton, MD, USA: Central Statistical Agency and ICF International: 2017.

[20] Ahmed HM, Mitchell M, Hedt B. National implementation of Integrated Management of Childhood Illness (IMCI): policy constraints and strategies. Health policy. 2010;96(2):128–33. 10.1016/j.healthpol.2010.01.013 20176407

[21] Gebretsadik. assessment of the prevalence and associated factors of pneumonia in children 2to 59 months old, debreberhan district, north east Ethiopia. 2015.

[22] Organization WH. Integrated Management of Childhood Illness: distance learning course. 2014.

[23] Kulldorff M. inventorSaTScan-Software for the Spatial. Temporal and Space-Time Scan Statistic. 2015.

[24] Alemu K, Worku A, Berhane Y, Kumie A. Spatiotemporal clusters of malaria cases at village level, northwest Ethiopia. Malaria journal. 2014;13(1):223. 24903061 10.1186/1475-2875-13-223 PMC4072611

[25] Teshome A. Prevalence of pneumonia and factors associated among children 2–59 months old in Wondo Genet district, Sidama zone, SNNPR, Ethiopia. Curr Pediatr Res. 2017;21(1):19–25.

[26] Ikeogu MO, Wolf B, Mathe S. Pulmonary manifestations in HIV seropositivity and malnutrition in Zimbabwe. Archives of disease in childhood. 1997;76(2):124–8. 9068301 10.1136/adc.76.2.124 PMC1717076

[27] Deribew A, Tessema F, Girma B. Determinants of under-five mortality in Gilgel Gibe Field Research Center, Southwest Ethiopia. Ethiopian Journal of Health Development. 2007;21(2):117–24.

[28] Schmidt W-P, Cairncross S, Barreto ML, Clasen T, Genser B. Recent diarrhoeal illness and risk of lower respiratory infections in children under the age of 5 years. International journal of epidemiology. 2009;38(3):766–72. 10.1093/ije/dyp159 19279073 PMC2689396

[29] Rahman SS, Khatun A, Azhar BS, Rahman H, Hossain S. A study on the relationship between nutritional status and prevalence of pneumonia and diarrhoea among preschool children in Kushtia. Pediatrics Research International Journal. 2014;2014:i1–10.

[30] Jabessa S. Multilevel Analysis of Acute Respiratory Infection Symptoms amo ng under Five Children in Ethiopia. Journal of Biometrics & Biostatistics. 2015;6(4):1.

[31] Fatmi Z, White F. A comparison of ‘cough and cold’and pneumonia: risk factors for pneumonia in children under 5 years revisited. International Journal of Infectious Diseases. 2002;6(4):294–301. 12718824 10.1016/s1201-9712(02)90164-5

[32] Ghimire M, Bhattacharya S, Narain J. Pneumonia in South-East Asia region: public health perspective. The Indian journal of medical research. 2012;135(4):459 22664492 PMC3385228

[33] Walker CLF, Rudan I, Liu L, Nair H, Theodoratou E, Bhutta ZA, et al Global burden of childhood pneumonia and diarrhoea. The Lancet. 2013;381(9875):1405–16. 10.1016/S0140-6736(13)60222-6 PMC7159282 23582727

[34] Andrade AL, Afonso ET, Minamisava R, Bierrenbach AL, Cristo EB, Morais-Neto OL, et al Direct and indirect impact of 10-valent pneumococcal conjugate vaccine introduction on pneumonia hospitalizations and economic burden in all age-groups in Brazil: A time-series analysis. PLoS One. 2017;12(9):e0184204 10.1371/journal.pone.0184204 28880953 PMC5589174

[35] Orellana JDY, Basta PC, Santos RV, Coimbra Junior CEA. Morbidade hospitalar em crianças indígenas Suruí menores de dez anos, Rondônia, Brasil: 2000 a 2004. 2007.

[36] Saha S, Hasan M, Kim L, Farrar JL, Hossain B, Islam M, et al Epidemiology and risk factors for pneumonia severity and mortality in Bangladeshi children< 5 years of age before 10-valent pneumococcal conjugate vaccine introduction. BMC public health. 2016;16(1):1233 10.1186/s12889-016-3897-9 27927201 PMC5142317

[37] Mishra P, Parajuli J, Acharya N, Gupta V. Malnutrition as a Modifiable Risk Factor of Lower Respiratory Tract Infections Among Under Five Children. Journal of Nepalgunj Medical College. 2016;12(2):2–5.

[38] Kirkwood BR, Gove S, Rogers S, Lob-Levyt J, Arthur P, Campbell H. Potential interventions for the prevention of childhood pneumonia in developing countries: a systematic review. Bulletin of the World Health Organization. 1995;73(6):793 8907773 PMC2486683

[39] Shibata T, Wilson JL, Watson LM, LeDuc A, Meng C, La Ane R, et al Childhood acute respiratory infections and household environment in an Eastern Indonesian urban setting. International journal of environmental research and public health. 2014;11(12):12190–203. 10.3390/ijerph111212190 25429685 PMC4276609

